# Clonal dominance of a donor‐derived del(20q) clone after allogeneic hematopoietic stem cell transplantation in an acute myeloid leukemia patient with del(20q)

**DOI:** 10.1002/jcla.22951

**Published:** 2019-06-11

**Authors:** Jung Yoon, Jae Won Yun, Chul Won Jung, Hee‐Jin Kim, Sun‐Hee Kim

**Affiliations:** ^1^ Department of Laboratory Medicine and Genetics, Samsung Medical Center Sungkyunkwan University School of Medicine Seoul Korea; ^2^ Department of Internal Medicine, Samsung Medical Center Sungkyunkwan University School of Medicine Seoul Korea

**Keywords:** 20q deletion, acute myeloid leukemia, age‐related clonal hematopoiesis, allogeneic hematopoietic stem cell transplantation

## Abstract

Del(20q) is the most frequently detected large structural genetic mosaicism alteration in the healthy aging population, occurring in approximately 0.1% of older persons. Age‐related clonal hematopoiesis of copy number variations (CNVs) is linked to an increased risk of hematologic malignancies, but the clinical impact of hematopoietic stem cells (HSCs) harboring such CNVs, such as del(20q), is not clearly understood. Here, we report an acute myeloid leukemia (AML) patient with known del(20q) who acquired donor‐derived del(20q) after allogeneic hematopoietic stem cell transplantation (HSCT). The HSCs with del(20q) engrafted and expanded over time, but the patient did not clinically progress to myeloid neoplasm. Although donor‐derived del(20q) from a healthy donor has been reported previously, our case is the first to review the clonal dynamics of a del(20q) clone and its post‐transplantation impact. Since recurrent hematology neoplasm‐associated CNVs, including del(20q), may not be rare among aged HSCT donors, identifying the origin of such a CNV is necessary for clinical decisions when clonal abnormality appears after HSCT, even in recipients who previously had the same abnormality.

## 1 INTRODUCTION

Deletion of 20q is a recurrent cytogenetic abnormality seen in a broad spectrum of hematologic malignancies. Del(20q) is observed in 5%‐7% of myelodysplastic syndrome (MDS), 1%‐2% of acute myeloid leukemia (AML), and 10% of myeloproliferative disease.^1^, ^2^, ^3^

Although frequently observed in myeloid neoplasm, del(20q) is insufficient as definitive evidence for diagnosis of myeloid neoplasm.^4^ Del(20q) has been suggested as an age‐related clonal hematopoiesis (ARCH),^5^ in that it was the most frequently detected large structural genetic mosaicism alteration in a healthy aging population.^6^ Moreover, the prevalence of 20q deletion was more common than that of myeloid neoplasm.^7^ ARCH‐related copy number variation (CNV) predicted an increased risk of hematologic malignancy,^8^, ^9^ but the clinical significance of hematopoietic stem cell transplantation (HSCT) from a donor with such clonal hematopoiesis remains uncertain.

Herein, we report the clonal dominance of a donor‐derived del(20q) clone after allogeneic HSCT in an AML patient with known del(20q) at diagnosis. No clinical or morphological progression to MDS or AML was evident, but clonal expansion and dominance of the del(20q) clone were noted at the most recent last 18‐month follow up.

## 2 MATERIAL AND METHODS

Conventional cytogenetics and fluorescence in situ hybridization (FISH).

Conventional karyotyping was analyzed in 24 hours without mitotic stimulation bone marrow (BM) aspirate samples. G‐banding was achieved using trypsin‐Wright staining, and the banded metaphases were described according to the International System for Hyman Cytogenetic Nomenclature (ISCN 2016).

Fluorescence in situ hybridization study was performed following the manufacturers’ instructions on interphase nuclei using the XL D20S108 SpectrumOrange/20qter SpectrumGreen Probe (MetaSystems); LSI MLL Dual Color, Break Apart Rearrangement Probe (Vysis); LSI TP53 SpectrumGreen/CEP17 SpectrumOrange Probe (Vysis); LSI D7S522 SpectrumOrange/D7Z1 SpectrumGreen Probe (Vysis); and LSI EGR1 SpectrumOrange/D5S23, D5S721 SpectrumGreen Probe (Vysis). A total of 200 interphase cells were evaluated.

## 3 RESULTS

**Figure 1:**
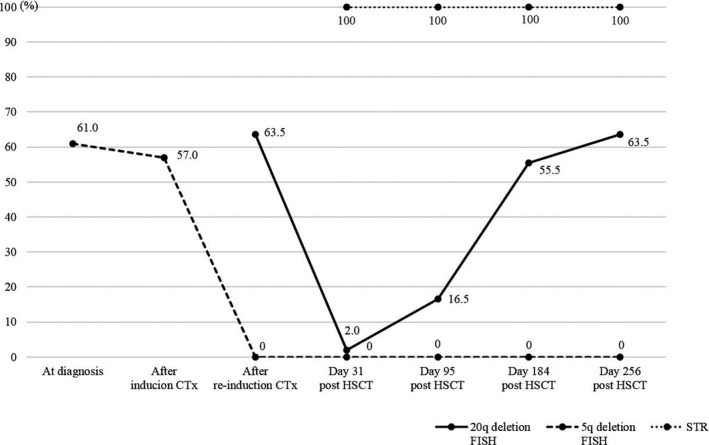
The clonal dynamics of del(20q) and del(5q) clones assessed by fluorescence in situ hybridization (FISH) after hematopoietic stem cell transplantation (HSCT). The short tandem repeat (STR) results are also displayed

A 57‐year‐old male was referred for general weakness and pancytopenia. Complete blood counts (CBCs) showed Hb 8.3 g/dL, WBC count 2020/μL, and PLT count 65 000/μL with 21% blasts. Bone marrow examination revealed increase of blasts up to 65% without evident dysplasia. Cytogenetic studies were performed with PB due to insufficient specimen and showed a normal male karyotype 46,XY in 20 metaphases analyzed. Fluorescence in situ hybridization analysis for *KMT2A* rearrangements, 17p deletion, 7q deletion, and 5q deletion identified 61.0% (122/200) of cells with loss of *EGR1* signal on 5q31. The patient was diagnosed with AML with myelodysplasia‐related changes (Figure 1).

The follow‐up BM examination after induction chemotherapy showed persistence of leukemic cells. Additional structural and numerical cytogenetic abnormalities including 20q deletion and reciprocal 5;12 translocation were observed in 3 of the 4 analyzable metaphases, with karyotype of 46,XY,der(5)t(5;12)(q31;q13),‐12,del(20)(q11.2q13.3),+mar[3]/46,XY[1]. Fluorescence in situ hybridization analysis showed persistent 5q deletion and 57.0% (114/200) of cells with a single *EGR1* signal (Figure 1).

After reinduction chemotherapy, BM biopsy showed no morphologic evidence of residual leukemic cells, confirmed by flow cytometry (<0.01% of total events). However, the cytogenetic study revealed discrepant results. Although a single *EGR1* signal in FISH analysis was not observed, del(20q) persisted in 16 of 20 metaphases. Fluorescence in situ hybridization study for 20q deletion was performed, and 63.5% (127/200) of cells showed a single D20S108 signal. Based on persistent cytogenetic abnormality of 20q deletion, diagnosis of morphologic complete remission was made (Figure 1).

The patient underwent HSCT from his HLA‐identical brother 3 months after achieving morphologic CR The donor was healthy with normal CBCs at the time of hematopoietic stem cell collection (HSCC). The patient responded well without any sign of GVHD. Neutrophils and platelets were engrafted by day 14 and day 21 (>50 × 10^9^/L), respectively. However, after achieving platelet recovery, the platelet count fluctuated, ranging from 20 × 10^9^/L to 50 × 10^9^/L. Follow‐up BM study performed 1 month later showed trilineage hematopoiesis without any residual leukemic cells or dysplasia but a decreased number of megakaryocytes. Post‐transplantation chimerism analysis using a short tandem repeat (STR) study demonstrated complete engraftment of donor cells. However, del(20q) persisted in 2 of 20 metaphases and FISH analysis results have showed 2% (4/200) of cells with a single D20S108 signal, which was below the positive cutoff value of 4% in our laboratory (Figure 1).

**Figure 2:**
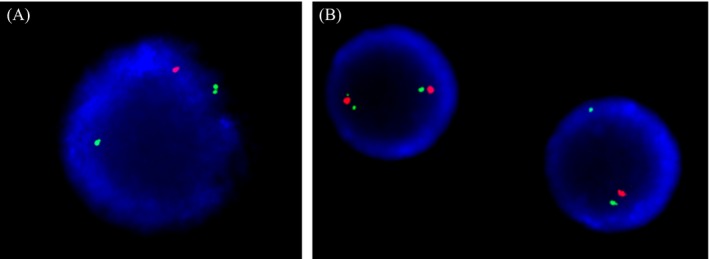
Fluorescence in situ hybridization analysis using a D20S108/20qter probe (XL D20S108 SpectrumOrange/20qter SpectrumGreen Probe, MetaSystems, Altlussheim, Germany). A, A single D20S108 signal was detected in up to 63.5% of cells in a bone marrow specimen 9 mo after hematopoietic stem cell transplantation. B, A single D20S108 signal was also detected in an aliquot of the hematopoietic stem cell collection

The last follow‐up BM study performed at 18 months after HSCT revealed decreased number of megakaryocytes along with decreased platelet count (ranging 20 × 10^9^/L‐50 × 10^9^/L), and the possibility of delayed platelet engraftment was suggested. The follow‐up cytogenetic studies performed using BM samples showed continuously increasing percentage of cells with del(20q) up to 15 of 20 metaphases with conventional cytogenetic study and to 63.5% (127/200) with FISH analysis, and the follow‐up STR study persistently demonstrated complete engraftment of donor cells (Figures 1 and 2). The patient remained healthy without any evidence of recurrence for over 30 months after HSCT. The platelet count gradually has increased up to 95 × 10^9^/L.

We performed FISH study for detection of del(20q) on an aliquot of HSCC to determine the clinical significance of reappeared del(20q); 8% of cells (16/200) from the HSCC showed the deletion (Figure 2). Taken together, these results suggest that the reappeared 20q deletion in our patient was donor derived.

## 4 DISCUSSION

Del(20q), along with other hematological malignancy‐associated CNVs such as 5q, 11q, and 17p, has been identified in 2% of healthy individuals older than 70 years. Such clonal hematopoiesis is an age‐related condition and predicts increased risk of hematologic malignancy.^8^, ^9^

Since del(20q) is asymptomatic, donor stem cells harboring del(20q) may be engrafted in the recipient during HSCT. The clinical impact of donor‐derived del(20q) as a part of clonal hematopoiesis is not clear. To our knowledge, there was one case of transmission of del(20q) from a healthy donor to chronic lymphocytic leukemia patient. Although the recipient died 3 months later due to severe graft‐vs‐host disease, the authors did not observe any evidence of progression to myeloid neoplasm. Like our case, clonal expansion of donor‐derived del(20q) was noted.^10^

In our case, defining the origin of reappeared del(20q) was crucial in that the patient was diagnosed with AML with del(20q) before undergoing HSCT. We were able to conclude the possibility of relapse to not likely after confirming the STR results showing complete chimerism and after identifying the del(20q) clone as donor derived. Several related questions have been raised. What is the fate of the del(20q) clone in our patient? Since the first case study with donor‐derived del(20q) followed the patient for a relatively short period of time, the outcome of our patient was not conclusive. Should del(20q) in our patient considered as the newly emerged isolated del(20q) in patients following cytotoxic therapies? The patients who acquired isolated del(20q) after cytotoxic therapy had innocuous BM finding in more than 77% of patients.^11^, ^12^ Moreover, would the abnormal clone react equivalently in our patient as in the donor? The interplay of BM microenvironment and leukemia cells has been suggested,^13^ and there is a chance that the del(20q) clone will progress to myeloid neoplasm in the BM microenvironment of someone previously diagnosed with AML harboring the same cytogenetic abnormality.

As del(20q) has been suggested as an ARCH, we presumed that donor‐derived del(20q) will share some features of donor‐derived clonal hematopoiesis of indeterminate potential (CHIP). The impact of donor‐derived CHIP in HSCT recipients is controversial. Among patients with impaired hematopoietic recovery after HSCT, CHIP was commonly found, and a strong association between unexplained cytopenia and HSCT from donors with CHIP has been suggested.^14^ Another recent study reviewed recipients allografted with donor CHIP and found a lower cumulative incidence of relapse/progression and no effect on overall survival despite the increased risk of donor cell leukemia.^15^ The impact of CHIP in overall survival did not vary between the underlying hematologic malignancies that led to HSCT, except myeloproliferative neoplasm. Both studies have shown that donor cell leukemia due to donor‐engrafted CHIP is a relatively rare complication.^14^, ^15^

Although few cases are present to confer the role of donor‐engrafted del(20q), all such (2/2) cases showed expansion of del (20q) clones,^10^ which is more frequent than in previously studied donor‐derived CHIP.^14^, ^15^ It is possible that del(20q) may have a more competitive advantage in hematopoietic stem cells than in CHIP, producing mature cells with del(20q) and contributing to clonal expansion.

Despite the clonal dominance of del(20q), the patient remains healthy without any evidence of myeloid neoplasm. The patient recovered to normal CBCs except for persistent thrombocytopenia. We believe that the thrombocytopenia in our patient was due to delayed engraftment, considering the decreased number of megakaryocytes. The association of del(20q) with delayed engraftment of a megakaryocyte lineage needs to be determined through further studies. Although clinical progression to overt myeloid neoplasm was not evident in our patient, a study with a larger sample is needed to clearly define the fate of the del(20q) clone and to rule out the possibility of clonal evolution with additional mutation acquisition.

In summary, our case showed that del(20q), one of the most common CNVs in healthy, aged individuals, did not clinically progress to myeloid neoplasm even in a patient with known AML‐harboring del(20q) clone. Since recurrent hematology neoplasm‐associated CNVs are not rare among aged HSCT donors, identifying the origin of such CNVs is necessary for clinical decisions regarding recipients with clonal abnormality appearing after HSCT. Clonal expansion was observed in a del(20q) clone, and a larger study with more patients is required to fully understand the fate of a del(20q) clone.

## CONFLICT OF INTEREST

The authors have no conflict of interest to declare.
